# Psychosomatic symptom profiles identified by latent profile analysis and their psychosocial and clinical correlates in patients with type 2 diabetes mellitus: A cross-sectional study

**DOI:** 10.1097/MD.0000000000048153

**Published:** 2026-04-17

**Authors:** He Yin, Haixia Ma, Xiaoju Zhang, Chao Li, Chunguang Wang, Qingqing Li

**Affiliations:** a The Nursing Department, Beijing University of Chinese Medicine Dongfang Hospital Qinhuangdao Hospital (Qinhuangdao Hospital of Traditional Chinese Medicine), Qinhuangdao, Hebei Province, China; b Beijing University of Chinese Medicine Dongfang Hospital Qinhuangdao Hospital (Qinhuangdao Hospital of Traditional Chinese Medicine), Qinhuangdao, Hebei Province, China; c Endocrinology Department, Beijing University of Chinese Medicine Dongfang Hospital Qinhuangdao Hospital (Qinhuangdao Hospital of Traditional Chinese Medicine), Qinhuangdao, Hebei Province, China.

**Keywords:** latent profile analysis, psychosomatic symptoms, quality of life, self-efficacy, social support, type 2 diabetes mellitus

## Abstract

Psychosomatic symptoms are highly prevalent among individuals with type 2 diabetes mellitus (T2DM), yet they often cluster into heterogeneous patterns that are inadequately characterized by single-symptom assessments or composite scores. This study aimed to identify latent psychosomatic symptom profiles in T2DM and to examine their associations with clinical characteristics, psychosocial resources, and health-related quality of life. This single-center cross-sectional study enrolled 312 adults with T2DM. Six psychosomatic symptom domains (depressive symptoms, anxiety, fatigue, sleep quality, pain intensity, and diabetes-related distress) were assessed as continuous indicators and analyzed using latent profile analysis. Sociodemographic and clinical variables, psychosocial resources (self-efficacy and perceived social support), and quality-of-life measures were compared across identified profiles. Multinomial logistic regression was conducted with the low symptom burden profile as the reference, using a clinical model (age, diabetes duration, and glycated hemoglobin) and an extended model incorporating psychosocial resources. Among the 312 participants (mean age 58.5 ± 10.3 years; 59.6% male), a 3-profile solution provided optimal fit (entropy = 0.93; Lo–Mendell–Rubin test *P* = .012). The profiles comprised a low symptom burden group (59.6%), a high fatigue–emotional distress group (21.8%), and a high overall symptom burden group (18.6%). Symptom severity differed significantly across profiles (all *P* < .001). Compared with the low symptom burden profile, individuals in the high symptom burden profile were older, had longer diabetes duration, and more frequently reported neuropathy and prior hypoglycemia (all *P* ≤ .05), whereas glycated hemoglobin and body mass index did not differ significantly. In multivariable analyses, greater self-efficacy (odds ratio 0.66, 95% confidence interval 0.56–0.78) and higher perceived social support (odds ratio 0.92, 95% confidence interval 0.89–0.95) were independently associated with reduced odds of belonging to the high symptom burden profile. Health-related quality of life demonstrated a clear gradient across profiles, with the poorest physical and mental health scores in the high symptom burden group (*P* < .001). Distinct psychosomatic symptom profiles were identified among patients with T2DM. A high symptom burden profile was characterized by greater clinical complexity, diminished psychosocial resources, and substantially impaired quality of life, independent of glycemic control.

## 1. Introduction

Type 2 diabetes mellitus is one of the most prevalent chronic metabolic disorders worldwide and is characterized by substantial clinical heterogeneity.^[1,2]^ Conventional management has long focused on glycemic control and the prevention of diabetes-related complications, yet patients with similar levels of glycemic control often report markedly different symptom experiences, functional limitations, and quality of life.^[3–6]^ Such discrepancies reflect an important clinical reality in which outcomes in type 2 diabetes cannot be fully explained by glycemic indices alone.^[7–11]^

Patients with type 2 diabetes frequently experience heterogeneous psychosomatic symptom burdens that are associated with treatment adherence, self-management capacity, and health-related quality of life.^[12,13]^ Depressive symptoms, anxiety, fatigue, sleep disturbances, chronic pain, particularly pain related to diabetic neuropathy, and diabetes-related distress are all highly prevalent in this population.^[14–16]^ These symptoms are not isolated clinical features but often emerge simultaneously, forming complex and interrelated patterns that contribute to an overall psychosomatic symptom burden.^[17–19]^ Despite this clinical reality, much of the existing literature has examined these symptoms in isolation or has relied on simple summed scores to represent overall symptom severity.^[20,21]^ Such approaches may obscure symptom heterogeneity and clustering, leading to misclassification of patients with similar scores but distinct symptom profiles and limiting the clinical relevance of symptom assessment.

Conventional variable-centered analyses, such as regression-based models, primarily estimate average associations between individual variables and clinical outcomes.^[22,23]^ While informative, these approaches may be insufficient in chronic disease populations characterized by substantial symptom heterogeneity, where the identification of distinct subgroups of patients is often more clinically relevant than population-level effects.^[11,24]^ Latent profile analysis (LPA) represents a person-centered analytic framework that uses multiple continuous symptom indicators to identify unobserved subgroups with relatively homogeneous symptom patterns.^[25,26]^ This approach has been successfully applied in research on chronic pain, cardiovascular conditions, and mental health disorders.^[27–32]^ However, studies employing LPA to systematically integrate multidimensional psychosomatic symptoms in patients with type 2 diabetes remain limited.^[22–24]^

Psychosocial resources, particularly self-efficacy and perceived social support, play an important role in the management of type 2 diabetes and are closely linked to patients’ capacity to cope with long-term disease demands.^[33–36]^ Higher self-efficacy has been associated with better self-management behaviors and lower emotional distress, while adequate social support may buffer disease-related stressors and symptom burden.^[37–39]^ However, these resources are most often examined as independent predictors of specific outcomes, rather than within a broader framework of symptom patterning.^[40,41]^ Given that psychosomatic symptom burden substantially affects daily functioning and quality of life, even among patients with comparable glycemic control, symptom burden appears to represent a distinct dimension of disease impact beyond traditional metabolic indicators.^[42,43]^ Identifying high-risk symptom profiles and their psychosocial correlates may therefore support more individualized assessment, targeted intervention strategies, and improved patient-reported outcomes in type 2 diabetes.

We hypothesized that distinct latent symptom profiles with varying levels of symptom burden would be observed, and that the high symptom burden profile would be characterized by poorer psychosocial resources and lower quality of life. Therefore, the present study aimed to identify latent psychosomatic symptom profiles among patients with type 2 diabetes using LPA based on multiple symptom dimensions. It further sought to compare sociodemographic characteristics, clinical features, psychosocial resources, and quality of life across the identified profiles, and to examine the independent associations of psychosocial resources with the high symptom burden profile.

## 2. Methods

### 2.1. Study design and participants

This study adopted a cross-sectional observational design. Participants were recruited from the endocrinology and chronic disease outpatient clinics and inpatient wards of Qinhuangdao Hospital of Traditional Chinese Medicine. Recruitment was conducted between January 2023 and December 2023, using a consecutive sampling approach. Adult patients diagnosed with type 2 diabetes mellitus who met the eligibility criteria were invited to participate. A total of 312 patients were included in the final analysis, comprising 186 men and 126 women.

The study protocol was reviewed and approved by the Ethics Committee of Qinhuangdao Hospital of Traditional Chinese Medicine (Approval No.: 2023-01-05). Written informed consent was obtained from all participants prior to enrollment. This cross-sectional study was conducted and reported in accordance with the Strengthening the Reporting of Observational Studies in Epidemiology guidelines.

### 2.2. Inclusion and exclusion criteria

Participants were eligible for inclusion if they met all of the following criteria: a confirmed diagnosis of type 2 diabetes mellitus based on established diagnostic criteria, as documented in the medical records. Age of 40 years or older at the time of enrollment, ensuring that the study population predominantly represented middle-aged and older adults with type 2 diabetes. Clinical stability, defined as the absence of acute diabetic emergencies or major acute medical events within the preceding 3 months, including but not limited to diabetic ketoacidosis, hyperosmolar hyperglycemic state, severe infection, or acute cardiovascular or cerebrovascular events. Ability to complete questionnaires, indicated by adequate cognitive and communicative capacity to understand the study procedures and to complete the psychosomatic symptom, psychosocial resource, and quality-of-life questionnaires, either independently or with minimal assistance from trained research staff. Willingness to participate in the study and provision of written informed consent prior to enrollment.

Participants were excluded if any of the following conditions were present: severe psychiatric disorders, defined as a current or previous diagnosis of serious psychiatric illness, such as schizophrenia, bipolar disorder in the manic phase, or other psychotic disorders, which could substantially interfere with reliable symptom assessment. Significant cognitive impairment, including moderate to severe cognitive dysfunction or dementia that precluded adequate understanding of the questionnaires or reliable self-reporting. Severe acute or terminal illness, defined as the presence of serious acute medical conditions or end-stage diseases, including advanced malignancy or organ failure, that could markedly influence psychosomatic symptom burden or quality-of-life assessment. Incomplete or invalid questionnaire data, characterized by substantial missing data in key questionnaires related to psychosomatic symptoms, psychosocial resources, or quality of life, exceeding a predefined threshold or rendering the data unsuitable for statistical analysis.

### 2.3. Sociodemographic and clinical variables

Sociodemographic information was collected using a structured questionnaire and included age (continuous), sex (male or female), educational attainment (≤9 years or >9 years), and marital status (married or unmarried, divorced, or widowed). Clinical variables related to type 2 diabetes mellitus were obtained through a combination of medical record review and participant self-report. These variables included duration of diabetes (years), body mass index (BMI, kg/m^2^), and glycated hemoglobin (HbA1c, %). HbA1c values were derived from the most recent laboratory assessment available at the time of enrollment. Diabetes treatment modality was categorized as lifestyle intervention only, oral hypoglycemic agents, or insulin therapy (alone or in combination with oral agents). A history of hypoglycemia within the preceding 6 months was recorded as a binary variable (yes or no). Diabetes-related complications were also documented, including the presence of any complication, with particular attention to diabetic peripheral neuropathy. Information on complications was obtained by integrating medical record documentation with patient self-report.

### 2.4. Psychosomatic symptom measures

Psychosomatic symptoms constituted the core input variables for LPA and were therefore assessed as a distinct domain. Six symptom dimensions were evaluated using validated self-report instruments, with all scores treated as continuous variables.

Depressive symptoms were assessed using the Patient Health Questionnaire-9, which measures the frequency of depressive symptoms over the previous 2 weeks, with higher total scores indicating more severe depressive symptoms. Anxiety symptoms were measured using the Generalized Anxiety Disorder-7 scale, in which higher scores reflect greater anxiety severity. Fatigue was evaluated using the Fatigue Severity Scale, which assesses the impact of fatigue on daily functioning. Sleep quality was assessed using the Pittsburgh Sleep Quality Index, with higher scores indicating poorer sleep quality. Pain intensity was measured using the numerical rating scale, ranging from 0 to 10, and was used to capture chronic pain, particularly pain related to diabetic peripheral neuropathy. Diabetes-related distress was assessed using the Diabetes Distress Scale, which reflects emotional distress specifically associated with diabetes management and its perceived burden.

All symptom scales demonstrated good internal consistency in the present sample. For all measures, higher scores indicated greater psychosomatic symptom burden.

### 2.5. Psychosocial resources

Psychosocial resources were assessed as potentially modifiable protective factors and were included in subsequent regression analyses to evaluate their incremental explanatory value. Self-efficacy was measured using a validated chronic disease self-efficacy scale, which reflects patients’ confidence in managing disease-related tasks and challenges. Perceived social support was assessed using the Perceived Social Support Scale or a comparable validated instrument, capturing the extent to which individuals perceive emotional and practical support from their social network. Higher scores on both scales indicated greater levels of psychosocial resources.

### 2.6. Quality of life

Quality of life was assessed using a validated generic or diabetes-specific quality-of-life instrument. The measure provided an overall quality-of-life score as well as subscale scores reflecting physical health and mental health domains. Higher scores indicated better perceived quality of life across all dimensions.

### 2.7. Statistical analysis

#### 2.7.1. Descriptive statistical analysis

Continuous variables were summarized as means with standard deviations, whereas categorical variables were presented as frequencies and percentages. Comparisons between groups were conducted as appropriate according to variable type and distribution. Independent-samples *t* tests or one-way analysis of variance were used to compare continuous variables across sex or latent profile groups. Categorical variables were compared using the Chi-square test. All analyses were performed using two-sided tests.

#### 2.7.2. Latent profile analysis

LPA was conducted to identify unobserved subgroups of patients based on psychosomatic symptom patterns. Six continuous symptom indicators were included in the LPA, namely depressive symptoms, anxiety symptoms, fatigue, sleep quality, pain intensity, and diabetes-related distress. Models specifying 1 to 5 latent profiles were sequentially estimated. Model fit and classification quality were evaluated using multiple statistical indices, including the Akaike information criterion (AIC), Bayesian information criterion, sample-size adjusted Bayesian information criterion, entropy, the Lo–Mendell–Rubin adjusted likelihood ratio test, and the bootstrap likelihood ratio test. The optimal number of latent profiles was determined by jointly considering model fit, classification accuracy, statistical significance of likelihood ratio tests, and model parsimony.

#### 2.7.3. Comparison of characteristics across latent profiles

After identification of the optimal latent profile solution, differences across profiles were examined to characterize the clinical relevance of each subgroup. Comparisons were conducted for psychosomatic symptom levels, sociodemographic and clinical characteristics, psychosocial resources, and quality of life. Continuous variables were compared using one-way analysis of variance, and categorical variables were compared using the Chi-square test. These analyses corresponded to the presentation of symptom patterns and profile-specific characteristics.

#### 2.7.4. Multinomial logistic regression analysis

Multinomial logistic regression analyses were performed to examine factors associated with latent profile membership. Latent profile membership was treated as the dependent variable, with the low symptom burden profile designated as the reference category. Independent variables were entered in a stepwise manner. Model A included age, duration of diabetes, and HbA1c as clinical covariates. Model B further incorporated psychosocial resources, including self-efficacy and perceived social support, to assess their incremental associations with latent profile membership. Odds ratios (ORs) with corresponding 95% confidence intervals (CIs) and *P* values were reported. These models were used to identify risk and protective factors associated with membership in the high symptom burden profile.

LPA was conducted using Mplus version 8.8 (Muthén & Muthén, Los Angeles). Models were estimated using robust maximum likelihood estimation, which is robust to non-normality. Missing data were handled using full information maximum likelihood under the assumption of missing at random.

Continuous indicator variables were specified with freely estimated means and variances across profiles, while indicators were assumed to be conditionally independent within each profile (i.e., within-profile covariances were fixed to zero). Models with increasing numbers of profiles were estimated sequentially.

To reduce the risk of local maxima, each model was estimated using 500 random sets of starting values with 100 final-stage optimizations. Model convergence was evaluated by replication of the best log-likelihood value. Model selection considered statistical fit indices (AIC, Bayesian information criterion [BIC], adjusted BIC), entropy, likelihood ratio tests, and clinical interpretability of the profiles.

Descriptive analyses and regression models were conducted using SPSS. LPA was performed using Mplus. All statistical tests were two-sided, and a *P* value of <.05 was considered to indicate statistical significance.

## 3. Results

### 3.1. Baseline characteristics of the study population

A total of 312 patients with type 2 diabetes mellitus were included in the analysis. Detailed baseline characteristics are presented in Table 1. The mean age of the study population was 58.47 ± 10.26 years, and 59.62% of participants were male. The average duration of diabetes was 8.73 ± 6.15 years. Overall, glycemic control was suboptimal, with a mean HbA1c level of 8.12 ± 1.64%, and 75.64% of participants had HbA1c levels ≥7.0%. The mean BMI was 26.38 ± 3.47 kg/m^2^.

**Table 1:** Baseline characteristics of the study population with type 2 diabetes mellitus.

Characteristic	Overall (n = 312)	Male (n = 186)	Female (n = 126)	*P* value
Age (yr)	58.47 ± 10.26	57.92 ± 10.14	59.28 ± 10.41	.214
Male sex, n (%)	186 (59.62)	–	–	–
Duration of diabetes (yr)	8.73 ± 6.15	8.61 ± 6.02	8.91 ± 6.34	.681
Body mass index (kg/m^2^)	26.38 ± 3.47	26.71 ± 3.39	25.89 ± 3.54	.032
HbA1c (%)	8.12 ± 1.64	8.05 ± 1.58	8.23 ± 1.72	.287
Poor glycemic control (HbA1c ≥ 7.0%), n (%)	236 (75.64)	138 (74.19)	98 (77.78)	.468
**Diabetes treatment regimen, n (%**)				.612
Lifestyle management only	22 (7.05)	14 (7.53)	8 (6.35)	
Oral antidiabetic drugs only	168 (53.85)	98 (52.69)	70 (55.56)	
Insulin only	54 (17.31)	32 (17.20)	22 (17.46)	
Oral agents + insulin	68 (21.79)	42 (22.58)	26 (20.63)	
History of hypoglycemia (past 6 months), n (%)	74 (23.72)	46 (24.73)	28 (22.22)	.612
**Any diabetes-related complication, n (%**)	168 (53.85)	102 (54.84)	66 (52.38)	.673
Diabetic neuropathy, n (%)	96 (30.77)	60 (32.26)	36 (28.57)	.482
Diabetic retinopathy, n (%)	74 (23.72)	42 (22.58)	32 (25.40)	.567
Diabetic nephropathy, n (%)	58 (18.59)	36 (19.35)	22 (17.46)	.662

Values are presented as mean ± standard deviation or number (percentage), as appropriate. *P* values were calculated using independent-samples *t* tests for continuous variables and χ^2^ tests for categorical variables. Poor glycemic control was defined as HbA1c ≥ 7.0%. History of hypoglycemia was defined as any self-reported or documented hypoglycemic episode within the past 6 months.

BMI = body mass index, HbA1c = glycated hemoglobin.

Regarding treatment regimens, more than half of the participants were treated with oral antidiabetic agents alone (53.85%), while 39.10% received insulin-based therapy, either alone or in combination with oral agents. A history of hypoglycemia within the past 6 months was reported in 23.72% of patients. More than half of the study population (53.85%) had at least 1 diabetes-related complication, with diabetic neuropathy being the most common (30.77%), followed by diabetic retinopathy (23.72%) and nephropathy (18.59%).

The distribution of psychosomatic symptom measures used for LPA is summarized in Table S1, Supplemental Digital Content, https://links.lww.com/MD/R589. In the overall sample, the mean scores indicated mild to moderate levels of depressive and anxiety symptoms, with Patient Health Questionnaire-9 and Generalized Anxiety Disorder-7 scores of 7.84 ± 5.26 and 6.92 ± 4.81, respectively. Fatigue was prevalent, with a mean Fatigue Severity Scale score of 4.38 ± 1.27. Sleep quality was generally impaired, as reflected by a mean Pittsburgh Sleep Quality Index score of 8.64 ± 3.92. The mean pain intensity score was 3.21 ± 2.34 on the numerical rating scale. Diabetes-related distress was also evident, with a mean Diabetes Distress Scale score of 2.46 ± 0.83.

### 3.2. Identification of latent psychosomatic symptom profiles

Latent profile models with 1 to 5 profiles were estimated and compared. As shown in Table 2, the AIC decreased from 10,234.58 for the 1-profile model to 9412.36 for the 3-profile model, with further reductions in the 4- and 5-profile solutions. Similar decreasing patterns were observed for the BIC (from 10,301.41–9558.68) and the adjusted BIC (from 10,248.17–9441.12).

**Table 2:** Fit indices for latent profile models of psychosomatic symptom measures (N = 312).

Number of profiles	AIC	BIC	aBIC	Entropy	LMRT *P* value	BLRT *P* value
1	10,234.58	10,301.41	10,248.17	–	–	–
2	9638.71	9745.28	9659.89	0.97	.001	<.001
3	9412.36	9558.68	9441.12	0.93	.012	<.001
4	9289.44	9475.50	9325.79	0.91	.084	<.001
5	9224.63	9450.43	9268.57	0.89	.263	<.001

Latent profile analysis (LPA) models with 1 to 5 profiles were estimated using 6 psychosomatic symptom indicators (depressive symptoms, anxiety symptoms, fatigue, sleep quality, pain intensity, and diabetes-related distress). Lower values of AIC, BIC, and adjusted BIC (aBIC) indicate better model fit. Entropy reflects classification precision, with higher values indicating clearer separation between profiles. The Lo–Mendell–Rubin adjusted likelihood ratio test (LMRT) and the bootstrap likelihood ratio test (BLRT) compare the fit of the k-profile model against the (k − 1)-profile model; a *P* value <.05 suggests the k-profile solution provides a statistically improved fit.

aBIC = adjusted Bayesian information criterion, AIC = Akaike information criterion, BIC = Bayesian information criterion.

Classification accuracy remained high, with entropy values of 0.97 for the 2-profile solution and 0.93 for the 3-profile solution. The Lo–Mendell–Rubin adjusted likelihood ratio test supported the 2-profile (*P* = .001) and 3-profile (*P* = .012) solutions, but not the 4-profile (*P* = .084) or 5-profile (*P* = .263) models. The bootstrap likelihood ratio test remained significant for all models (*P* < .001).

**Figure 1.**
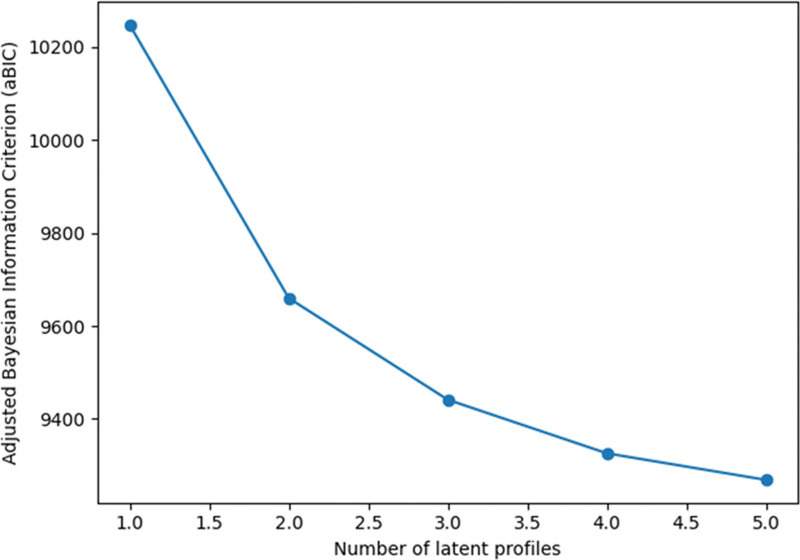
Adjusted Bayesian information criterion (aBIC) values for latent profile models. Adjusted Bayesian information criterion (aBIC) values for latent profile models with 1 to 5 profiles. The aBIC values decreased substantially from the 1-profile to the 3-profile solution, followed by a more gradual decline with additional profiles, indicating diminishing improvements in model fit beyond 3 profiles. The inflection point at the 3-profile solution supports its selection as the optimal model.

Inspection of the adjusted BIC curve showed a pronounced decline from the 1-profile to the 3-profile solution, followed by a more gradual decrease thereafter, with a clear inflection point at 3 profiles (Fig. 1). Accordingly, the 3-profile solution was selected as the optimal model.

Based on this solution, 186 participants (59.62%) were classified into Profile 1, 68 (21.79%) into Profile 2, and 58 (18.59%) into Profile 3.

### 3.3. Symptom patterns and characterization of each latent profile

**Figure 2.**
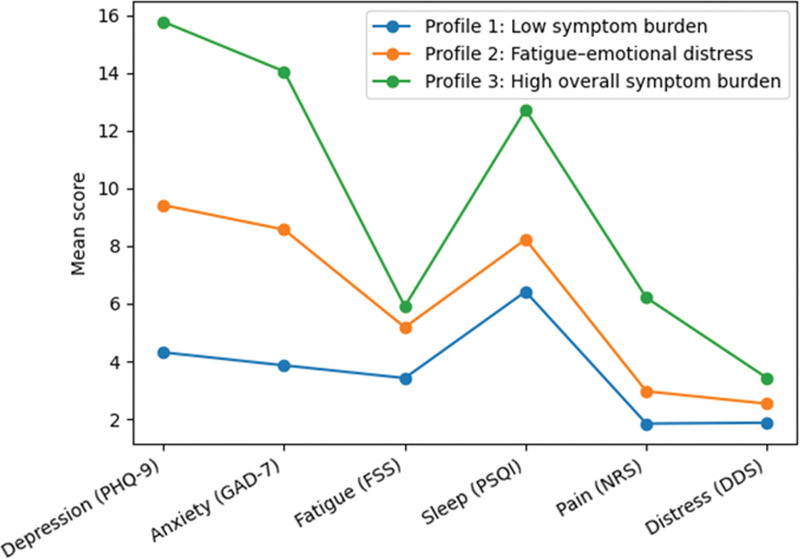
Psychosomatic symptom patterns across latent profiles. Mean scores of depressive symptoms (PHQ-9), anxiety symptoms (GAD-7), fatigue severity (FSS), sleep quality (PSQI), pain intensity (NRS), and diabetes-related distress (DDS) are shown for each latent profile. Higher scores indicate greater symptom burden. GAD-7 = Generalized Anxiety Disorder-7, NRS = numerical rating scale, PHQ-9 = Patient Health Questionnaire-9, PSQI = Pittsburgh Sleep Quality Index.

As shown in Table 3 and Figure 2, the 3 latent profiles demonstrated distinct psychosomatic symptom patterns (all *P* < .001). Profile 1 (low psychosomatic symptom burden) exhibited consistently low levels across depressive and anxiety symptoms, fatigue, sleep disturbance, pain intensity, and diabetes-related distress, representing a reference-like group. Profile 2 (high fatigue–emotional distress) was characterized by prominent fatigue accompanied by elevated depressive and anxiety symptoms, whereas sleep disturbance, pain, and diabetes-related distress were comparatively moderate. Profile 3 (high overall psychosomatic symptom burden) showed uniformly high scores across all symptom domains, including markedly worse sleep quality, higher pain intensity, and substantially elevated diabetes-related distress, representing the highest-burden subgroup. Bonferroni-adjusted pairwise comparisons confirmed significant differences between latent profiles across psychosomatic symptom indicators (Table S2, Supplemental Digital Content, https://links.lww.com/MD/R589).

**Table 3:** Psychosomatic symptom measures across latent profiles (N = 312).

Measure	Profile 1 (n = 186)	Profile 2 (n = 68)	Profile 3 (n = 58)	*P* value
Depressive symptoms (PHQ-9 score)	4.31 ± 2.74	9.42 ± 3.96	15.78 ± 5.12	<.001
Anxiety symptoms (GAD-7 score)	3.86 ± 2.61	8.57 ± 3.42	14.06 ± 4.63	<.001
Fatigue severity (FSS score)	3.42 ± 0.84	5.18 ± 0.93	5.91 ± 0.88	<.001
Sleep quality (PSQI score)	6.42 ± 2.87	8.23 ± 2.94	12.73 ± 3.31	<.001
Pain intensity (NRS score)	1.84 ± 1.62	2.96 ± 2.04	6.21 ± 1.78	<.001
Diabetes-related distress (DDS score)	1.87 ± 0.46	2.53 ± 0.62	3.42 ± 0.68	<.001

Values are presented as mean ± standard deviation. *P* values indicate overall differences across latent profiles and were obtained using one-way analysis of variance (ANOVA). Higher scores indicate greater symptom severity or distress for all measures.

DDS = Diabetes Distress Scale, FSS = Fatigue Severity Scale, GAD-7 = Generalized Anxiety Disorder-7, NRS = numerical rating scale, PHQ-9 = Patient Health Questionnaire-9, PSQI = Pittsburgh Sleep Quality Index.

### 3.4. Differences in sociodemographic and clinical characteristics across latent profiles

Sociodemographic characteristics differed across latent profiles (Table 4). Participants in Profile 3 were older than those in Profiles 1 and 2 (62.14 ± 10.76 vs 56.92 ± 9.84 and 59.38 ± 10.21 years; *P* = .002). Lower educational attainment (≤9 years) was more common in Profile 3 (58.62%) than in Profile 1 (39.78%; *P* = .018), while the proportion of married participants decreased across profiles (81.72% in Profile 1 vs 65.52% in Profile 3; *P* = .021). Sex distribution did not differ significantly (*P* = .083). Diabetes duration increased from Profile 1 to Profile 3 (7.42 ± 5.36–11.28 ± 6.58 years; *P* < .001). In contrast, HbA1c and BMI were similar across profiles (*P* = .124 and *P* = .318). Diabetes-related complications were more prevalent in Profiles 2 and 3 than in Profile 1 (61.76% and 68.97% vs 46.24%; *P* = .006), with a marked gradient for diabetic neuropathy (22.58%, 35.29%, and 51.72%, respectively; *P* < .001). A history of hypoglycemia and insulin-based therapy were more common in higher-burden profiles (*P* = .008 and *P* = .015).

**Table 4:** Sociodemographic and clinical characteristics across latent psychosomatic symptom profiles.

Characteristic	Profile 1 (n = 186)	Profile 2 (n = 68)	Profile 3 (n = 58)	*P* value
Age (yr)	56.92 ± 9.84	59.38 ± 10.21	62.14 ± 10.76	.002
Male sex, n (%)	118 (63.44)	38 (55.88)	30 (51.72)	.083
Education ≤ 9 years, n (%)	74 (39.78)	32 (47.06)	34 (58.62)	.018
Married, n (%)	152 (81.72)	50 (73.53)	38 (65.52)	.021
Duration of diabetes (yr)	7.42 ± 5.36	9.61 ± 6.04	11.28 ± 6.58	<.001
HbA1c (%)	7.96 ± 1.52	8.21 ± 1.63	8.37 ± 1.79	.124
Body mass index (kg/m^2^)	26.12 ± 3.31	26.74 ± 3.58	26.89 ± 3.66	.318
**Any diabetes-related complication, n (%**)	86 (46.24)	42 (61.76)	40 (68.97)	.006
Diabetic neuropathy, n (%)	42 (22.58)	24 (35.29)	30 (51.72)	<.001
History of hypoglycemia (past 6 months), n (%)	34 (18.28)	18 (26.47)	22 (37.93)	.008
Insulin-based therapy, n (%)	62 (33.33)	32 (47.06)	28 (48.28)	.015

Values are presented as mean ± standard deviation or number (percentage), as appropriate. *P* values were calculated using one-way analysis of variance (ANOVA) for continuous variables and χ^2^ tests for categorical variables. Insulin-based therapy refers to insulin alone or in combination with oral antidiabetic agents.

BMI = body mass index, HbA1c = glycated hemoglobin.

### 3.5. Psychosocial resources across latent profiles

Psychosocial resources differed significantly across latent profiles (Table 5). Self-efficacy scores showed a clear decreasing trend from Profile 1 to Profile 3 (7.42 ± 1.18, 6.38 ± 1.24, and 5.41 ± 1.36, respectively; *P* < .001), with the lowest levels observed in the high symptom burden profile. Similarly, perceived social support declined progressively across profiles, with mean scores of 63.28 ± 9.46 in Profile 1, 56.71 ± 10.14 in Profile 2, and 48.92 ± 11.27 in Profile 3 (*P* < .001).

**Table 5:** Psychosocial resources across latent psychosomatic symptom profiles.

Measure	Profile 1 (n = 186)	Profile 2 (n = 68)	Profile 3 (n = 58)	*P* value
Self-efficacy score	7.42 ± 1.18	6.38 ± 1.24	5.41 ± 1.36	<.001
Perceived social support score	63.28 ± 9.46	56.71 ± 10.14	48.92 ± 11.27	<.001

Values are presented as mean ± standard deviation. *P* values were obtained using one-way analysis of variance (ANOVA). Higher scores indicate greater self-efficacy and stronger perceived social support.

### 3.6. Factors associated with latent profile membership

In multinomial logistic regression analyses (Table 6), Model A showed that age and diabetes duration were independently associated with higher odds of membership in the high symptom burden profile (age: OR = 1.06, 95% CI 1.03–1.09, *P* < .001; duration: OR = 1.10, 95% CI 1.06–1.14, *P* < .001). HbA1c was not significantly associated with latent profile membership (OR = 1.14, 95% CI 0.99–1.32, *P* = .064).

**Table 6:** Multinomial logistic regression models for factors associated with latent profile membership (N = 312).

Variable	Profile 2 vs 1 OR (95% CI) Model A	*P* value	Profile 2 vs 1 OR (95% CI) Model B	*P* value	Profile 3 vs 1 OR (95% CI) Model A	*P* value	Profile 3 vs 1 OR (95% CI) Model B	*P* value
Age (per yr)	1.03 (1.00–1.05)	.036	1.02 (0.99–1.04)	.124	1.06 (1.03–1.09)	<.001	1.04 (1.01–1.07)	.004
Duration of diabetes (per yr)	1.05 (1.02–1.08)	.002	1.04 (1.01–1.07)	.011	1.10 (1.06–1.14)	<.001	1.08 (1.04–1.12)	<.001
HbA1c (%)	1.09 (0.96–1.24)	.184	1.07 (0.94–1.22)	.303	1.14 (0.99–1.32)	.064	1.10 (0.95–1.28)	.201
Self-efficacy score	–	–	0.84 (0.74–0.95)	.007	–	–	0.66 (0.56–0.78)	<.001
Perceived social support score	–	–	0.96 (0.94–0.99)	.003	–	–	0.92 (0.89–0.95)	<.001

Reference group: Profile 1 (low psychosomatic symptom burden). Odds ratios (ORs) and 95% confidence intervals (CIs) were estimated using multinomial logistic regression, with Profile 1 (low psychosomatic symptom burden) as the reference category. Model A included age, duration of diabetes, and HbA1c. Model B additionally included self-efficacy and perceived social support. ORs >1 indicate increased odds of membership in the corresponding profile; ORs <1 indicate decreased odds.

CI = confidence interval; HbA1c = glycated haemoglobin; OR = odds ratio.

After additional adjustment for psychosocial resources in Model B, age and diabetes duration remained significantly associated with the high symptom burden profile, although effect estimates were attenuated (age: OR = 1.04, 95% CI 1.01–1.07, *P* = .004; duration: OR = 1.08, 95% CI 1.04–1.12, *P* < .001). Higher self-efficacy (OR = 0.66, 95% CI 0.56–0.78, *P* < .001) and greater perceived social support (OR = 0.92, 95% CI 0.89–0.95, *P* < .001) were independently associated with lower odds of membership in the high symptom burden profile. HbA1c remained nonsignificant (OR = 1.10, 95% CI 0.95–1.28, *P* = .201).

### 3.7. Differences in quality of life across latent profiles

Quality of life differed significantly across latent profiles (Table 7). Overall quality of life scores decreased progressively from Profile 1 to Profile 3 (72.84 ± 10.26, 64.37 ± 11.42, and 53.91 ± 12.78, respectively; *P* < .001). Similar graded declines were observed in both physical health (74.12 ± 11.03, 65.48 ± 12.26, and 54.28 ± 13.34; *P* < .001) and mental health domains (71.36 ± 10.87, 63.19 ± 11.98, and 52.74 ± 12.91; *P* < .001). Patients in the high symptom burden profile exhibited the poorest quality of life.

**Table 7:** Quality of life across latent psychosomatic symptom profiles.

Quality of life measure	Profile 1 (n = 186)	Profile 2 (n = 68)	Profile 3 (n = 58)	*P* value
Overall quality of life score	72.84 ± 10.26	64.37 ± 11.42	53.91 ± 12.78	<.001
Physical health domain	74.12 ± 11.03	65.48 ± 12.26	54.28 ± 13.34	<.001
Mental health domain	71.36 ± 10.87	63.19 ± 11.98	52.74 ± 12.91	<.001

Values are presented as mean ± standard deviation. *P* values were obtained using one-way analysis of variance (ANOVA). Higher scores indicate better quality of life across all domains.

## 4. Discussion

Using a person-centered analytic approach based on multiple psychosomatic symptoms, this study identified 3 distinct and well-differentiated latent symptom profiles among patients with type 2 diabetes. These findings highlight pronounced heterogeneity and clustering of psychosomatic symptoms within this population. The high symptom burden profile was independently associated with older age, longer diabetes duration, and poorer psychosocial resources. In contrast, glycemic control, as reflected by HbA1c levels, did not adequately account for membership in this profile. Importantly, patients classified into the high symptom burden profile exhibited substantially impaired quality of life. Together, these results underscore that psychosomatic symptom burden represents a clinically meaningful dimension of disease impact beyond glycemic indices alone.

The use of LPA enabled the identification of distinct psychosomatic symptom patterns that are unlikely to be captured by conventional variable-centered approaches.^[44–47]^ This analysis revealed clinically interpretable subgroups characterized by different constellations and levels of symptom burden, rather than extremes along a single dimension.^[48–51]^ Notably, the presence of an intermediate profile with selective symptom elevation suggests that psychosomatic burden in type 2 diabetes follows differentiated patterns rather than a simple low–high continuum.^[22,23,52–56]^ Overall, these findings underscore the value of LPA in capturing clinically meaningful heterogeneity in symptom experience and informing subsequent patient-centered analyses.

Previous studies in type 2 diabetes have largely focused on individual symptoms, such as depression, anxiety, or pain, or have relied on aggregated symptom scores to quantify overall burden.^[35,57–61]^ While these approaches have provided important insights, they offer limited resolution for capturing the complex and heterogeneous ways in which psychosomatic symptoms co-occur at the individual level.^[36,57,61,62]^ By contrast, the present study simultaneously integrated multiple psychosomatic symptom dimensions and applied a person-centered LPA to identify distinct patient subgroups. In addition, the incorporation of psychosocial resources and quality of life extends prior work by linking symptom patterns to modifiable contextual factors and patient-centered outcomes. Rather than challenging existing evidence, this approach builds on the current literature by offering a more nuanced framework for patient stratification in type 2 diabetes.^[11,26,63–66]^

The identified latent profiles appeared to differ primarily in overall symptom severity rather than reflecting sharply distinct symptom configurations. Profile 1 was characterized by generally low levels across psychosomatic indicators, whereas Profiles 2 and 3 showed progressively higher symptom burden across domains. This pattern suggests a severity-graded structure of psychosomatic distress among patients with type 2 diabetes rather than discrete symptom-specific phenotypes. Such gradients may still carry clinical relevance by identifying patients with increasing levels of psychosomatic burden requiring differentiated levels of support.

Patients classified into the high psychosomatic symptom burden profile exhibited a distinct pattern of clinical and sociodemographic vulnerability. This group tended to be older, had lower educational attainment, and reported less marital or social support, along with a longer duration of diabetes. Diabetes-related complications, particularly peripheral neuropathy, as well as a history of hypoglycemia were more prevalent among these patients. Notably, no significant differences were observed in HbA1c levels or BMI across symptom profiles. Taken together, this pattern suggests that psychosomatic symptom burden reflects cumulative clinical and psychosocial vulnerability rather than glycemic control alone.

Psychosocial resources, particularly self-efficacy and perceived social support, demonstrated a clear graded decline across latent symptom profiles, with the lowest levels observed in the high symptom burden group. In multinomial logistic regression analyses, both self-efficacy and social support remained independently associated with lower odds of membership in the high symptom burden profile, even after adjustment for age, diabetes duration, and HbA1c. Notably, the inclusion of psychosocial resources in the extended model modestly attenuated the associations of clinical variables, while the overall direction and significance of these associations remained stable. This pattern highlights the incremental explanatory value of psychosocial resources beyond traditional clinical factors.^[34,57–59,67]^ From a clinical perspective, these findings suggest that a high psychosomatic symptom burden does not necessarily represent an immutable condition and that psychosocial resources may constitute a feasible and actionable entry point for supportive interventions in patients with type 2 diabetes.

Clear and graded differences in quality of life were observed across the identified symptom profiles, with a progressive decline from the low to the high psychosomatic burden group. Individuals with higher symptom burden reported substantially poorer physical and mental health–related quality of life, indicating that psychosomatic burden reflects meaningful variation in patients’ lived experiences rather than merely statistical classification. These findings suggest that psychosomatic symptom patterns represent an important dimension of patient well-being that extends beyond traditional metabolic indicators. From a clinical perspective, symptom-based profiling may help contextualize patient experiences and support more comprehensive assessment of psychosocial needs alongside standard diabetes management. However, given the cross-sectional design, these implications should be interpreted as hypothesis-generating rather than causal.

Consistent with diabetes-specific literature demonstrating associations between psychosocial distress and poorer self-management and patient-reported outcomes, psychosomatic burden may help identify individuals who could benefit from enhanced psychosocial support and multidisciplinary care approaches.^[68–70]^ Importantly, the identified profiles primarily reflected gradients of overall symptom burden rather than distinct phenotypes; therefore, LPA should be viewed as a complementary, person-centered framework for summarizing multidimensional distress rather than a superior clinical classification method. Interpretation of self-efficacy also warrants caution, as its relationship with outcomes may depend on contextual and structural factors. Accordingly, the present findings indicate associative relationships between psychosocial resources and symptom burden profiles, and future longitudinal and interventional studies are required to evaluate causal pathways and clinical utility.

Several limitations should be acknowledged. The cross-sectional design prevents inference regarding causality, directionality, or temporal stability of symptom profiles, which should be regarded as cross-sectional classifications rather than stable subtypes. In addition, the single-center sample may limit generalizability across healthcare systems and cultural contexts. Psychosomatic symptoms and psychosocial resources were assessed using self-reported measures, which may introduce reporting bias and shared-method variance, and objective indicators were not included. Future multicenter longitudinal studies integrating behavioral and biological measures are needed to evaluate profile stability, external validity, and the clinical implications of psychosomatic burden stratification in type 2 diabetes.

## 5. Conclusion

In this study, 3 distinct psychosomatic symptom profiles were identified among patients with type 2 diabetes using a person-centered analytic approach. The high symptom burden profile was characterized by greater clinical and psychosocial vulnerability and was associated with substantially impaired quality of life. Psychosocial resources, particularly self-efficacy and perceived social support, emerged as important protective factors associated with lower odds of belonging to the high symptom burden profile, highlighting their potential clinical relevance. Collectively, these findings support a more comprehensive, patient-centered approach to diabetes management beyond glycemic control alone.

## Acknowledgments

The authors sincerely thank all study participants for their invaluable contributions.

## Author contribution

**Conceptualization:** He Yin, Haixia Ma, Xiaoju Zhang, Chao Li, Chunguang Wang, Qingqing Li.

## Supplementary Material


