# Supplementary material for: Psychosomatic symptom profiles identified by latent profile analysis and their psychosocial and clinical correlates in patients with type 2 diabetes mellitus: A cross-sectional study
Source: Medicine (Baltimore). 2026 Apr 17;105(16):e48153. doi: 10.1097/MD.0000000000048153 (PMC13095260; doi:10.1097/MD.0000000000048153)
Supplement: Supplementary file 1 [file medi-105-e48153-s001.docx]

**Supplementary Table S1. Distribution of Psychosomatic Symptom Measures in the Overall Study Population**

| **Measure** | **Overall (n = 312)** |
| --- | --- |
| **Depressive symptoms (PHQ-9 score)** | 7.84 ± 5.26 |
| **Anxiety symptoms (GAD-7 score)** | 6.92 ± 4.81 |
| **Fatigue Severity Scale (FSS score)** | 4.38 ± 1.27 |
| **Sleep quality (PSQI score)** | 8.64 ± 3.92 |
| **Pain intensity (NRS score)** | 3.21 ± 2.34 |
| **Diabetes-related distress (DDS score)** | 2.46 ± 0.83 |

Values are presented as mean ± standard deviation.
PHQ-9 = Patient Health Questionnaire-9; GAD-7 = Generalized Anxiety Disorder-7;
FSS = Fatigue Severity Scale; PSQI = Pittsburgh Sleep Quality Index;
NRS = Numerical Rating Scale; DDS = Diabetes Distress Scale.
Higher scores indicate greater symptom severity or distress for all measures.

**Supplementary Table S2. Bonferroni-Adjusted Pairwise Comparisons of Psychosomatic Symptom Measures Across Latent Profiles (N = 312)**

| **Measure** | **Comparison** | **Mean Difference** | **Standard Error** | **Adjusted P value** |
| --- | --- | --- | --- | --- |
| **Depressive symptoms (PHQ-9)** | Profile 1 vs Profile 2 | −5.11 | 0.56 | <0.001 |
|  | Profile 1 vs Profile 3 | −11.47 | 0.63 | <0.001 |
|  | Profile 2 vs Profile 3 | −6.36 | 0.71 | <0.001 |
| **Anxiety symptoms (GAD-7)** | Profile 1 vs Profile 2 | −4.71 | 0.52 | <0.001 |
|  | Profile 1 vs Profile 3 | −10.20 | 0.59 | <0.001 |
|  | Profile 2 vs Profile 3 | −5.49 | 0.66 | <0.001 |
| **Fatigue severity (FSS)** | Profile 1 vs Profile 2 | −1.76 | 0.14 | <0.001 |
|  | Profile 1 vs Profile 3 | −2.49 | 0.15 | <0.001 |
|  | Profile 2 vs Profile 3 | −0.73 | 0.17 | 0.002 |
| **Sleep quality (PSQI)** | Profile 1 vs Profile 2 | −1.81 | 0.43 | 0.001 |
|  | Profile 1 vs Profile 3 | −6.31 | 0.49 | <0.001 |
|  | Profile 2 vs Profile 3 | −4.50 | 0.54 | <0.001 |
| **Pain intensity (NRS)** | Profile 1 vs Profile 2 | −1.12 | 0.29 | 0.004 |
|  | Profile 1 vs Profile 3 | −4.37 | 0.31 | <0.001 |
|  | Profile 2 vs Profile 3 | −3.25 | 0.35 | <0.001 |
| **Diabetes-related distress (DDS)** | Profile 1 vs Profile 2 | −0.66 | 0.08 | <0.001 |
|  | Profile 1 vs Profile 3 | −1.55 | 0.09 | <0.001 |
|  | Profile 2 vs Profile 3 | −0.89 | 0.10 | <0.001 |

Values represent Bonferroni-adjusted pairwise comparisons following significant omnibus one-way ANOVA tests. Mean differences are calculated as the first profile minus the second profile. Negative values indicate higher symptom severity in the comparison profile listed second.

Bonferroni correction was applied to control for multiple comparisons.

PHQ-9 = Patient Health Questionnaire-9;
GAD-7 = Generalized Anxiety Disorder-7;
FSS = Fatigue Severity Scale;
PSQI = Pittsburgh Sleep Quality Index;
NRS = Numerical Rating Scale;
DDS = Diabetes Distress Scale.

Higher scores indicate greater symptom severity or distress for all measures.
